# HCC and Molecular Targeting Therapies: Back to the Future

**DOI:** 10.3390/biomedicines9101345

**Published:** 2021-09-28

**Authors:** Luca Rinaldi, Erica Vetrano, Barbara Rinaldi, Raffaele Galiero, Alfredo Caturano, Teresa Salvatore, Ferdinando Carlo Sasso

**Affiliations:** 1 Department of Advanced Medical and Surgical Sciences, University of Campania Luigi Vanvitelli, Piazza Luigi Miraglia 2, I-80138 Naples, Italy; luca.rinaldi@unicampania.it (L.R.); erica.vetrano@unicampania.it (E.V.); raffaele.galiero@unicampania.it (R.G.); alfredo.caturano@unicampania.it (A.C.); 2 Department of Experimental Medicine, Section of Pharmacology, University of Campania Luigi Vanvitelli, Piazza Luigi Miraglia 2, I-80138 Naples, Italy; barbara.rinaldi@unicampania.it; 3 Department of Precision Medicine, University of Campania Luigi Vanvitelli, Via De Crecchio 7, I-80138 Naples, Italy; teresa.salvatore@unicampania.it

**Keywords:** liver, HCC, drugs, trials

## Abstract

Hepatocellular carcinoma (HCC) is one of the leading causes of death from cancer in the world. Recently, the effectiveness of new antiviral therapies and the HBV vaccine have reduced HCC’s incidence, while non-alcoholic steato-hepatitis is an emerging risk factor. This review focuses on antiangiogenic molecules and immune checkpoint inhibitors approved for HCC treatment and possible future approaches. Sorafenib was the first drug approved for the treatment of advanced HCC (aHCC) and it has been shown to increase survival by a few months. Lenvatinib, a multikinase inhibitor, has shown non-inferiority in survival compared with sorafenib and an improvement in progression-free survival (PFS). The combination of atezolizumab (an anti-PDL1 antibody) and bevacizumab (an anti-VEGF antibody) was the first drug combination approved for HCC, demonstrating improved survival compared with sorafenib (19.2 vs. 13.4 months). As a second line of therapy, three regimens (regorafenib, cabozantinib, and ramucirumab) have been approved for the treatment of aHCC after progression on sorafenib according to guidelines. Furthermore, nivolumab, pembrolizumab, and nivolumab plus ipilimumab have been approved by the FDA (2017, 2018, and 2020, respectively). Finally, immune target therapy, cancer vaccines, and epigenetic drugs represent three new possible weapons for the treatment of HCC.

## 1. Introduction

The most frequent liver cancer, and seventh by type of cancer in the world, is hepatocellular carcinoma (HCC), which is commonly associated with chronic hepatitis B virus (HBV) and hepatitis C virus (HCV) infections that usually develop during the cirrhosis stage [1,2,3,4]. HBV integrates into the genome and has a recognized carcinogenic action, while HCV does not integrate into the genome, but can induce epigenetic changes that can disregulate oncogenes [5,6]. Vaccination for HBV and new antiviral therapies that limit or clear the viral load reduce the risk of HCC and all hepatic and extrahepatic viral complications [5,7]. However, special conditions persist that require the monitoring of these patients [7,8,9,10]. In fact, the eradication of HCV with direct-acting antivirals (DAAs) does not delete the HCC risk and the histological picture of cirrhosis and the possible interference of the DAA with the genome can maintain a residual risk [6]. The presence of occult HBV can also represent a potential carcinogenic stimulus [11]. In addition to viruses, alcohol abuse, metabolic liver disease, and obesity represent other important risk factors for HCC. While in a condition of alcohol abuse the pathophysiological evolution from alcoholic cirrhosis to the development of HCC is well understood, the relation between a dysmetabolic condition and HCC appears to be much more complex. Non-alcoholic fatty liver disease (NAFLD) currently represents the most frequent manifestation of chronic liver disease [12]. The development of non-alcoholic steato-hepatitis (NASH) is an element of possible progression to cirrhosis with an increased risk of HCC [13,14]. Host genetic variants, especially the gene coding for patatin-like phospholipase domain-containing 3 (PNPLA3), may play a role in the development of HCC independently of activity and the extent of liver damage [15]. Two important elements may delay the diagnosis of HCC: many cases of HCC develop in patients with NAFLD in the absence of cirrhosis; and, secondly, people do not consider NAFLD to be as dangerous as viral liver infections [16]. Recently, the link between metabolic syndrome and liver diseases has been highlighted even more with the definition of metabolic-associated liver diseases (MALDs) [17]. Insulin resistance seems to be the connecting element between the diseases and underlies the development of type 2 diabetes (T2D) [18,19]. This latter is burdened with numerous complications and is associated with an increased risk of HCC in patients with NASH cirrhosis [20,21,22,23,24,25,26]. Transcription factors such as Kruppel-like factor 6, abnormal methylation, and immune dysregulation might help to explain the dysregulation of nine hub genes that have been identified as possible links between these two diseases [27].

The diagnosis of HCC is generally made through standard ultrasound with a contrast medium, which in the surveillance phase allows for early detection of small lesions [28]. Transient elastography using fibroscan represents a support method capable of monitoring some populations at greatest risk of HCC [29]. The diagnosis of HCC is confirmed with second-level methods and a histological biopsy that represents the gold standard [29,30].

The therapeutic strategies of HCC are limited by the patient’s basal clinical conditions. The coexistence of cirrhosis is an important limitation already burdened by complications such as portal hypertension and liver failure [31,32]. Whenever possible, selective surgical resection is the ideal method for eradicating the disease with a good expectation in terms of survival [33]. Alternatively, loco-regional treatments, such as radiofrequency, microwave, laser, and trans-arterial chemoembolization (TACE) treatments, allow us to obtain good results in terms of efficacy with limited damage for the most fragile patients. Liver transplantation can be considered in younger patients in order to obtain a synergistic action on HCC and the underlying disease, especially under particular conditions represented by an early stage of disease and favorable cancer biology, which offers excellent survival expectations [34,35,36,37]. However, constant monitoring of the patient and adherence to immunosuppressive therapy remain essential [38,39].

The failure or inability to carry out interventional eradication therapies orientates the therapeutic strategy to the use of drugs.

Until a few years ago, the options were very limited. However, more recently, numerous drugs have been tested, some of which have been approved for use in clinical practice in specialized centers. Moreover, several ongoing clinical trials of new molecules could potentially expand the range of choices in the coming years.

In this review, we describe the drugs currently approved and in use for the medical therapy of HCC, the main molecules studied in ongoing clinical trials, and novel targets for treatment. We do not describe drug therapies for NASH and associated cirrhosis.

## 2. Drugs Approved for HCC

Several new substances have changed the field of treatment for patients with HCC. Initially, no effective therapy was available after the failure of loco-regional approaches; however, in 2007 a new age started with the approval of sorafenib as the first effective systemic agent in patients with advanced HCC (aHCC). However, it took nearly 10 years for new and effective drugs to be used in both first-line and subsequent treatment. Since their recent approval, these new substances have changed the field of palliative treatment strategies for patients with aHCC, and their sequential application has been shown to be able to significantly prolong patient survival in the palliative approach. Recently, molecular targeted therapy has emerged as a new strategy of cancer treatment and, compared with traditional therapies, operates more specifically by destroying cancer cells, reducing damage to normal tissues, and being safer and better tolerated by patients [40,41]. Several studies detected dozens of mutations and driver genes with high frequency that could be considered to be the origin of HCC. Altered CTNNB1 is commonly found in HCC (23–36%) and is linked to WNT-β-catenin signaling. Active CTNNB1 mutations are more common in hepatitis C virus (HCV)-related HCC (more than half of HCV patients) than in hepatitis B virus (HBV)-related HCC and are associated with a particular WNT gene expression profile [42]. VEGFA is another driver gene in HCC (frequency: 7–10%) and mostly detected as copy number alterations [41]. Furthermore, a high level of VEGFA in HCC cells could lead to excessive production of hepatocyte growth factor (HGF), which induces tumor cell proliferation. KRAS (rat sarcoma of Kirsten), an isoform of RAS, is an oncogene that is frequently mutated in most cancers, although the mutation rate in HCC is relatively low (about 1%) [43]. Given the variety of mutations identified in a given patient, it is unlikely to have a therapeutic agent that effectively targets the majority of HCCs, thus requiring a combination of treatments to target different mutations [44]. Targeted molecular therapy acts on overexpressed cell receptors, key genes, and certain tumor cell marker molecules by selecting specific blockers to inhibit tumor growth, progress, and metastasis [40,45]. It is well known that, at any stage of HCC, vascular endothelial cell proliferation is active and the expression of VEGFR molecules on the cell surface is significantly upregulated [46]. Angiogenesis in cancer tissues has a major impact on the biological invasion capabilities of the cancer [47]. Therefore, blocking VEGF/VEGFR and reducing angiogenesis in tissues are considered to be new ideas for targeted therapy in HCC. Many molecularly targeted drugs have, both commercially and investigationally, achieved significant results. To date, based on phase III studies, six systemic therapies have been approved (atezolizumab plus bevacizumab, sorafenib, lenvatinib, regorafenib, cabozantinib, and ramucirumab) and three additional therapies have received accelerated Food and Drug Administration (FDA) approval due to evidence of efficacy. These drugs target the VEGFR-2 signal at various levels together with other receptors involved in the angiogenic process, with the exception of ramucirumab, which selectively targets VEGFR-2, so all these agents could be synergistically associated with immune checkpoint inhibitors [48]. Moreover, new studies are exploring drug combinations, including checkpoint inhibitors and tyrosine kinase inhibitors or anti-VEGF drugs, and even combinations of two immunotherapy regimens.

## 3. First-Line Therapy

### 3.1. The Progenitor: Sorafenib 

Sorafenib is a multikinase inhibitor that inhibits angiogenesis and tumor proliferation by interfering with the binding of serine/threonine kinases to receptor tyrosine kinases and acts on both cancer cells and endothelial cells [49,50]. It has been approved by the FDA for the treatment of unresectable HCC since 2007, based on the results of the SHARP and ORIENTAL trials, and since 2006 for the treatment of advanced renal cell carcinoma [51]. A dosage of 800 mg/day of sorafenib has demonstrated a survival benefit and it is the first-line systemic therapy for patients with progressive HCC [52]. Sorafenib primarily targets serine/threonine kinases, vascular endothelial growth factor receptor (VEGFR), platelet-derived growth factor receptor beta (PGFRβ), kit, fms-like tyrosine kinase-3 (FLT3), proto-oncogene ret (RET), and other receptor tyrosine kinases, which subsequently inhibit cancer cell proliferation and angiogenesis through mitogen-activated protein kinase (MAPK)/extracellular-signal-regulated kinase (ERK) [53]. In 602 patients with HCC who had received no previous systemic treatment, the median survival time in the sorafenib group was 2.8 months longer than in the placebo group (44%) [53]. Sorafenib treatment has been shown to provide a survival benefit in all subgroups of patients with HCC; however, the magnitude of the benefit was greater in patients with liver-confined hepatocellular carcinoma (without extrahepatic spread), in patients with HCV, and in patients with a lower neutrophil-to-lymphocyte ratio, an indicator of inflammatory status [54]. Wang et al. demonstrated that sorafenib, as an adjuvant therapy for liver cancer, can prevent early recurrence after hepatectomy [55,56]. In phase II studies, hepatic arterial infusion chemotherapy plus sorafenib showed favorable cancer control and a manageable safety profile, although in phase III studies it may have produced conflicting results [57]. However, sorafenib was found to be ineffective as an adjuvant treatment after curative resection or as a concomitant treatment with TACE [58,59,60]. The addition of hepatic arterial infusion chemotherapy to sorafenib did not significantly improve the overall survival in patients with aHCC [55]. During sorafenib treatment, associated toxicities, including gastrointestinal upset, anorexia, hand–foot skin reactions, and fatigue, were observed with an overall incidence of 30% and required permanent discontinuation in approximately 28% of treated patients [61].

### 3.2. The New First-Line Drugs

Since the approval of sorafenib in 2007, several new effective drugs have been established as a second-line treatment after progression with sorafenib, and more effective drugs have also been established for the first-line setting. A global open-label randomized phase III trial (REFLECT) demonstrated the efficacy of lenvatinib, which was the first new first-line drug approved for the treatment of aHCC in more than 10 years [62]. Lenvatinib is a VEGFR1-3, FGFR1-4, PDGFRα, tyrosine-Kinase RET receptor, and KIT receptor inhibitor [63]. The multi-center phase III REFLECT study demonstrated that lenvatinib was non-inferior to sorafenib in terms of overall survival in unresectable HCC [62]. A total of 954 patients with aHCC in Asia-Pacific, European, and North American regions were enrolled in the study, and the results showed that the median overall survival (mOS) was 13.6 months (95% CI, 12.1–14.9) in the lenvatinib group and 12.3 months (10.4–13.9; hazard ratio (HR), 0.92; 95% CI, 0.79–1.06) in the sorafenib group, meeting noninferiority criteria and suggesting that the survival benefits of lenvatinib were not inferior to those of sorafenib. Although the difference in overall survival in the lenvatinib group and the sorafenib group did not reach statistical significance, the progression-free survival (PFS) of the lenvatinib group was twice that of the sorafenib group and the time to progression was almost 3 times longer than in the sorafenib group [64]. A multi-center analysis reported that lenvatinib can be used safely and effectively regardless of age in patients with HCC. It was also seen that lenvatinib is not inferior to sorafenib in the first-line treatment of aHCC, especially in patients with HBV-related HCC [41,65]. In the REFLECT study, 83% of Asian patients had been infected with hepatitis B virus (HBV). Among patients with HBV-related HCC, the effective rate in the lenvatinib group was 21.5%, which was 2.6-fold higher than in the sorafenib group (8.3%) [62]. Cost–benefit analysis showed that lenvatinib offered a similar clinical efficacy at a lower cost than sorafenib, suggesting that lenvatinib would be a cost-effective alternative in the treatment of unresectable HCC [66]. A preclinical study showed that lenvatinib has more potent anti-tumor activity when combined with PD-1 inhibition, decreasing the number of tumor-associated macrophages and influencing anti-tumor immune responses [28]. Since both reduced and increased immunosuppression can result from blocking the VEGF/VEGFR axis, the combination of antiangiogenics and immune checkpoint inhibitors may represent an evolution of current treatment options [67]. In the randomized phase III non-inferiority trial, the overall incidence of adverse events was similar between the two treatment groups and the most common were hypertension (42%), diarrhoea (39%), decreased appetite (34%), and decreased weight (31%) for lenvatinib and palmar–plantar erythron dysesthesia (52%), diarrhoea (46%), hypertension (30%), and decreased appetite (27%) for sorafenib [67]. The combination of atezolizumab (an anti-PDL1 antibody) and bevacizumab (an anti-VEGF antibody) was the first regimen to improve overall survival compared with sorafenib [68]. Atezolizumab acts as an immunomodulator, blocking the ligand of the programmed cell death protein known as PD-L1. More specifically, atezolizumab blocks the interaction between PD-L1 and PD-1. PD-L1 can be highly expressed in certain tumor types, which, due to its interaction with the PD-1 protein, can reduce or even eliminate the proliferation of immune cells invading the cancer. Inhibition of PD-L1 therefore achieves the opposite effects: normal proliferation and infiltration of the tumor by immune cells and increased activity of the immune system [40,41,46]. Bevacizumab binds to vascular endothelial cell growth factor (VEGF), a protein that promotes angiogenesis and is present on the surface of blood vessels. Binding of the drug to VEGF prevents the latter from binding to its receptors (VEGFR-1 and VEGFR-2) on the surface of endothelial cells. By blocking the biological activity of VEGF, bevacizumab reverses the formation of new blood vessels and vascularization of the tumor, thus preventing cancer growth [68]. The IMbrave150 trial, an open-label study with patients randomized to sorafenib or to a combination of atezolizumab and bevacizumab as a first-line therapy for aHCC, demonstrated an improvement in overall survival with the combination therapy. An updated analysis shows that the median survival of patients receiving sorafenib was 13.4 months and the median survival of the combination arm was 19.2 months. The PFS was improved from 4.3 months in the sorafenib arm to 6.8 months in the combination arm, the Response Evaluation Criteria in Solid Tumors (RECIST) overall response rate (ORR) was increased from 11% in the sorafenib arm to 30% in the combination arm, and the median duration of response for the combination arm was 18.1 months by RECIST 1.1 and 16.3 months by RECIST 19 [68]. Patient-reported outcomes were also favorable to the combination arm, with the median time to deterioration of quality of life being 11.2 months compared with 3.6 months for sorafenib. Tolerability was more favorable in the combination group compared with sorafenib, with hypertension, proteinuria, and low-grade diarrhoea as the most common side effects. The autoimmune events that occurred with atezolizumab were reported as manageable. Upper gastrointestinal endoscopies were required within 6 months prior to enrolment for the treatment of varices in all patients to mitigate the risk of bleeding associated with bevacizumab. This timing of upper gastrointestinal endoscopies performed prior to treatment represents a change, especially for the screening of patients for the first-line therapy. Therefore, atezolizumab plus bevacizumab has become the standard of care in first-line therapies for aHCC, except in patients with untreated varices or in those with contraindications for VEGF inhibitors or immunotherapy [69].

## 4. Second-Line Therapies 

Based on positive phase III data and according to guidelines, three regimens (regorafenib, cabozantinib, and ramucirumab) have been approved for the treatment of aHCC after progression on sorafenib. Furthermore, based upon promising phase Ib/II studies, three additional therapies, namely nivolumab, pembrolizumab, and nivolumab plus ipilimumab, have been approved by the FDA after first-line treatment with sorafenib [70,71,72]. Regorafenib is an oral tyrosine kinase inhibitor (TKI) approved for patients with treatment-refractory metastatic colorectal cancer (mCRC), advanced gastrointestinal stromal tumor (GIST) after imatinib and sunitinib, and as a second-line drug in unresectable hepatocellular carcinoma (HCC) after sorafenib [73,74]. Approvals for GIST and HCC were based on the results of the randomized, placebo-controlled GRID and RESORCE phase 3 trials, respectively [75]. The molecular structures and mechanisms of action of regorafenib and sorafenib are very similar, but regorafenib has higher biological activity than sorafenib. Regorafenib inhibits kinases related to angiogenesis and tumorigenesis, such as VEGFR 1–3, the tyrosine receptor protein kinase Tie, RET, PDGFR, basic fibroblast growth factor receptor (FGFR), the serine/threonine protein kinase RAF, mitogen-activated protein kinase, and p38 kinase, thus playing an anti-tumoral role [76]. In the RESORCE study, regorafenib was tested as a second-line drug in 573 patients with HCC who had been treated with sorafenib, 194 of whom received a placebo [77]. The findings showed that, compared with the placebo group, regorafenib significantly improved the overall patient survival time (7.8 months in the placebo group vs 10.6 months in the experimental group). In two of the regorafenib-treated patients, the cancer shrank to an undetectable state [78]. An exploratory analysis of predictive biomarkers suggested an association between plasma protein and microRNA expression patterns on overall survival in patients with HCC after regorafenib treatment in RESORCE. The results showed that the benefit of regorafenib treatment for overall survival and prolonged median time to progression was independent of alpha-fetoprotein and c-Met levels [79]. In the RESORCE study, the most common drug-related adverse events of any grade were hand–foot skin reaction (HFSRs) (52%), diarrhoea (33%), fatigue (29%), anorexia (24%), and hypertension (23%). Clinically relevant grade ≥ 3 drug-related toxicities included hypertension (13%), HFSR (13%), hyperbilirubinemia (7%), fatigue (6%), and increased aspartate aminotransferase (5%) [77]. In addition to the approved uses of regorafenib in mCRC, GIST, and HCC following failure of standard therapies, there is a growing body of evidence demonstrating the efficacy of regorafenib in other cancer types. A clinical trial of regorafenib in various malignancies, including sarcomas and advanced biliary cancer, is ongoing [80]. Novel approaches are also being tested to refine and optimize regorafenib dosing for certain patient groups to improve tolerability while maintaining efficacy.

Cabozantinib is an effective multi-receptor TKI that can target VEGFR-2, c-Met, Kit, Axl, and FLT3. In the second-line phase III CELESTIAL trial, cabozantinib significantly improved overall survival in patients with liver cancer and was approved for use in patients with inoperable liver cancer [81]. Exposure to cabozantinib at the approved daily dose of 60 mg was seen to provide longer overall survival and a reduction in the rate of cancer progression or death, but an increase in adverse events compared with the initial doses of 40 mg or 20 mg. A subsequent dose reduction appeared to reduce risks of adverse events [82]. However, a cost-effectiveness analysis reported that cabozantinib at its current cost would not be cost-effective for patients with sorafenib-resistant HCC in the United States, United Kingdom, or China [82].

Ramucirumab is a recombinant IgG1 monoclonal antibody and VEGFR-2 antagonist. By blocking VEGF2, it inhibits cancer neoangiogenesis and curbs cancer growth. In a randomized, placebo-controlled, double-blind, multi-center, phase III trial (REACH), 565 patients were enrolled from 154 centers in 27 countries (283 were assigned to ramucirumab with 8 mg/kg every 2 weeks and 282 were assigned to a placebo). The result showed that the mOS was 9.2 months (95% CI, 8.0–10.6) in the ramucirumab group and 7.6 months (HR, 0.87; 95% CI, 0.72–1.05; *P* = 0.14) in the placebo group. Thus, the second-line treatment with ramucirumab did not significantly improve survival over the placebo in patients with aHCC [83]. Although the OS between the two groups was not statistically significant, subgroup analysis underlined that patients with elevated serum alpha fetoprotein (>400 ng/mL) achieved a better OS benefit from ramucirumab treatment compared with the placebo. The mOS in the ramucirumab group was 7.8 months, which was significantly greater than the 4.2 months in the placebo group. Accordingly, a randomized, double-blind, placebo-controlled, phase III trial (REACH-2) was initiated in patients with aHCC and increased α-fetoprotein concentrations [84]. In the REACH-2 study, 292 patients were enrolled in 20 countries and randomly assigned (197 to the ramucirumab group and 95 to the placebo group). The mOS was 8.5 months (95% CI, 7.0–10.6) in the ramucirumab group and 7.3 months in the placebo group (HR, 0.710; 95% CI, 0.53–0.95; *P* = 0.019) and the PFS was 2.8 months vs. 1.6 months (0.452; *P* < 0.0001). This study suggested that second-line treatment with ramucirumab significantly improved overall survival in HCC patients with a higher α-fetoprotein level of at least 400 ng/mL. In addition, ramucirumab was well tolerated with a manageable safety profile and a low incidence of adverse events. However, ramucirumab is not a cost-effective treatment from a United States payer perspective [85]. The main characteristics of the above trials are shown in Table 1.

## 5. FDA-Approved Drugs and Ongoing Trials

Based on data from phase Ib/II studies, nivolumab and pembrolizumab (anti-PD1 inhibitors) were approved as single agents, while ipilimumab (a CTLA4 monoclonal antibody) was approved in combination with nivolumab [71,72]. Nivolumab is an immune checkpoint inhibitor of programmed cell death protein 1 (PD-1). PD-1 is a co-inhibitory receptor expressed by activated T lymphocytes, which is necessary to evade immune surveillance. Blocking this protein stimulates the immune response and mediates tumor regression. The phase II CheckMate 040 study showed that patients receiving nivolumab had a relatively good safety profile [70]. According to the study, response to therapy lasted at least 6 months in 91% of responders and at least 12 months in 55% of responders. Nivolumab was granted accelerated FDA approval as the second-line treatment in aHCC based on the results of this study.

Pembrolizumab, a PD-1 monoclonal antibody, is an IgG4 that was evaluated in a phase II clinical trial in patients with aHCC after first-line therapy (Keynote-224) [86]. This study showed a median PFS of 4.8 months (95% CI, 3.4–6.6) with 6-month PFS and OS rates of 43.1% and 77.9%, respectively. Disease control was observed in 64 (62%; 95% CI, 52–71) of the 104 treated subjects, while among the 18 responders, 12 (77%) resulted in a response for at least 9 months [72]. Pembrolizumab was granted accelerated approval by the FDA as a second-line treatment for aHCC. In KEYNOTE-240, a phase III trial that studied pembrolizumab versus a placebo, 413 patients were randomized. The study did not show an improvement in mOS: the OS was 13.9 months (95% CI, 11.6–16.0 months) in the pembrolizumab arm and 10.6 months (95% CI, 8.3–13.5 months) in the placebo group (HR, 0.78; one sided *P* = 0.0238). PFS was not significantly improved by pembrolizumab, reaching 3 months with the treatment versus 2.8 months with the placebo (HR, 0.78; one sided *P* = 0.0209) [87].

The combination of nivolumab and ipilimumab achieved an objective response of 31% with a median duration of response of 17 months and a mOS of 23 months. Although the combination regimen induced immune-related toxicities requiring systemic corticoid administration in 51% of cases, the efficacy of outcomes resulted in accelerated approval by the FDA as a second-line therapy.

After the promising results of the new combination strategies, a non-randomized, open-label, multi-center, phase II study (RESCUE) investigated the association of an anti-PD-1 monoclonal antibody, camrelizumab, and a VEGFR-2 tyrosine kinase inhibitor, apatinib, in patients with aHCC who were treatment-naive or refractory or intolerant to first-line targeted therapy [88]. In particular, camrelizumab, an IgG4κ-humanized monoclonal antibody, targets programmed cell death protein 1 (PD-1), a protein on the surface of cells, that binds two ligands, programmed death-ligand 1 (PD-L1) and programmed death-ligand 2 (PD-L2). These interactions pharmacologically prevent the PD-1/PD-L1 interaction, thus facilitating a positive immune response to kill the tumor. The results of this phase II study demonstrated that camrelizumab plus apatinib showed a high ORR, promising efficacy, a long survival time, and manageable safety in aHCC patients.

Tislelizumab, another anti-PD-1 antibody, engineered to have a high affinity and binding specificity for PD-1, will be tested in a phase III randomized, open-label, multicenter clinical trial (RATIONALE 301) as a first-line treatment in patients with unresectable HCC in comparison to sorafenib [89]. This monoclonal antibody, as an antagonist to PD-L1/PD-L2 signaling, induced an increase in cytokine production and a restoration of T-cell activation, resulting in immune-mediated tumor cell death. In this phase III study, approximately 640 patients will be randomized, 1:1, to tislelizumab 200 mg intravenously every 3 weeks or sorafenib 400 mg orally twice daily and the primary objective is to compare OS between the two treatment groups. Positive results could add a new immunotherapy for unresectable HCC.

## 6. Combined Therapies

Based on the activity of single-agent immune checkpoint inhibitors (ICIs) and on a better understanding of the tumor immunosuppressive microenvironment (TME), several combination strategies can be considered and many of them have already entered into clinical development. The FDA, EMA, and other regulatory agencies worldwide have approved the atezolizumab plus bevacizumab combination for first-line therapy in HCC. This combination will therefore set a new standard of care for treatment-naive patients. Combinations result in a consistent twofold increase in response rates, with about 5% of patients in complete remission and long survival times of more than 18 months. In parallel, additional toxicities from combinations increase the number of serious adverse events leading to treatment discontinuation. ICIs have shown promising activity when paired with anti-angiogenic agents, other molecularly targeted therapies, and complementary ICIs. The VEGF pathway promotes local immune suppression through the inhibition of antigen-presenting cells and effector cells as well as through the activation of suppressive elements, including Treg cells, myeloid-derived suppressor cells, and tumor-associated macrophages, providing the rationale for combining ICIs with anti-angiogenic agents [90].

A phase Ib trial of the combination of lenvatinib and pembrolizumab as a first-line therapy in 100 unresectable patients with HCC demonstrated durable PFS [91]. Based upon the unique immunomodulatory and antiangiogenic profile of cabozantinib, another phase III trial to determine the efficacy of the combination of cabozantinib and atezolizumab compared with sorafenib or cabozantinib alone is ongoing [92,93]. 

From preliminary findings with the combination of ipilimumab and nivolumab, the best median mOS (22.8 months) was obtained with the highest dose (3 mg/kg once every 6 weeks) of the former and a lower dose of the latter (1 mg/kg once every 2 weeks) [71]. Such encouraging results have led to accelerated approval of this combination by the FDA to treat patients with HCC after sorafenib.

A similar effect was observed with the combination of a single 300 mg dose of tremelimumab combined with a continuous dose of the PDL1 inhibitor durvalumab. Tremelimumab is a fully human monoclonal antibody that binds to the CTLA-4 molecule. CTLA4 is expressed on the surface of activated T lymphocytes and, by binding to CTLA4, tremelimumab mediates downregulation of T-cell activation and then the immunitary response. Durvalumab is an IgG1 monoclonal antibody that binds with high affinity to the PD-L1 receptor and shows the same mechanism of action as atezolimumab. Interestingly, this single, high priming dose of tremelimumab resulted in an early burst of proliferating CD8+ T cells in peripheral blood [94]. These findings are in line with observations in melanoma, indicating that the activity of CTLA4 inhibitors is dose-dependent and that the first doses of CTLA4 inhibitors cause a proliferative burst of CD4+ and CD8+ T cells, probably related to the increased efficacy of the combination [95,96]. In HCC, as for other cancer types, combination regimens increase the rate of treatment-related adverse events (TRAEs) that are nevertheless tolerable.

In patients with unresectable HCC, a phase Ib study showed that lenvatinib plus pembrolizumab has promising anti-tumor activity; the mOS was 22 months and toxicities were manageable, with no unexpected safety signals [91].

An important question in the evaluation of the efficacy of a combination regimen is to understand whether improvements in time-to-event medians and objective response rates are due to synergy and not because of the independent additive effects of two active agents, which can also be achieved by a sequential approach [97]. In the absence of head-to-head trials or established biomarkers to guide the choice of therapy, treatment decisions must rely upon the magnitude of benefits, the toxicity profile, and drug availability. Biomarker data to help decision-making and to guide treatment for advanced stages of HCC are limited. An elevated level of serum α-fetoprotein is an established biomarker of poor prognosis across all stages of HCC and is associated with tumor VEGF pathway activation [98]. Serum levels of α-fetoprotein became the first biomarker predictive of response, with the finding of a survival benefit of ramucirumab over a placebo only in patients with α-fetoprotein levels ≥400 ng/mL [99]. Thus, ramucirumab is only indicated when α-fetoprotein levels are beyond this cut-off value. However, unlike ramucirumab, the treatment benefits from multi-kinase inhibitors, including sorafenib, lenvatinib, regorafenib, and cabozantinib, occur across a range of baseline α-fetoprotein values, likely owing to a broader spectrum of target inhibition on patients with elevated α-fetoprotein levels at baseline. Changes in α-fetoprotein levels on treatment were shown to correlate with clinical outcomes on systemic therapy, with declining α-fetoprotein levels linked to prolonged PFS and overall survival and increasing α-fetoprotein levels associated with tumor progression [100]. A variety of biomarkers that benefit from immune-checkpoint inhibition are under investigation across different solid tumors, including HCC. A meta-analysis of outcomes from >3500 patients showed that tumor PDL1 expression is associated with a worse prognosis in HCC, including a poorly differentiated histology, high levels of α-fetoprotein, and shorter overall survival [101]. The tumor lymphocytic infiltration immune class gene signature and CTNNB1 mutation status in subsets of HCC tumors also warrant examination for predictive value in patients treated with ICIs [102,103,104]. Unfortunately, no single biomarker was able to select HCC patients likely to benefit from immunotherapy, and the identification of predictors of response is an urgent and challenging need in this setting.

It would be very interesting, especially in the case of HCC post NAFLD, to evaluate the mechanistic assumptions and the possible clinical indication of the association of standard therapy with metformin, which in several other neoplastic conditions has shown an effect of enhancing therapies, especially in the second-line treatment [105]. The main characteristics of the above trials are reported in Table 2. 

## 7. The Next Level: Novel Targets for Treatment

Immune-targeted therapy could be considered a novel paradigm in the treatment of solid tumors, including aHCC [106]. Recent clinical data have underlined that HCC patients with a high number of cytotoxic T lymphocytes showed a significantly low risk of recurrence and a better prognosis [106]. Among these immunomodulatory approaches to HCC malignancy are cancer vaccines [107].

GPC3-targeted chimeric antigen receptor T cells (CAR-T cells) have been under investigation for the past few years in HCC patients [108]. In particular, the latest study on a GPC3 peptide vaccine, as an adjuvant therapy for HCC patients, was a phase II, open-label, single arm clinical trial [109]. In this study, forty-one patients with initial HCC who had undergone surgery or radiofrequency ablation were enrolled. The results showed that GPC3 peptide vaccination improves 1 and 2-year recurrence rates in GPC3-positive patients by 24.4% and 53.7%, respectively, compared with patients who received surgery only. Therefore, GPC3 is an important biomarker for clinical detection but is not able to induce a complete regression of the cancer. The next therapeutic option could be the realization of antigen cocktail vaccines targeting different tumor-associated antigens expressed in HCC, such as α-fetoprotein, Forkhead Box M1, and Wilms’ tumor-1.

The involvement of neoantigens (HANs) in the triggering of anti-tumor immunotherapy in HCC patients is a therapeutic strategy that has recently been investigated [110]. In particular, the authors of the study analyzed 56 needle biopsies and blood samples of HCC patients and demonstrated a correlation between the value of the HAN and a significant improvement in overall survival through the activation of tumor-reactive CD39+CD8+ T cells. In fact, the clinical results of the study showed that the 5-year survival rate of patients in the HAN-high group (60.76%, *p* = 0.0199) was better than that of patients in the HAN-low group (38.76%, *p* = 0.0199).

Despite the clinical progress of HANs in cancer therapy, their immunogenicity is low. It is therefore necessary to perform more optimized studies to identify epitopes with high immunogenicity.

An emerging option in cancer immunotherapy is represented by oncolytic viruses [111]. These virus particles are engineered to have a tropism for cancer cells and, by exerting a cytolytic effect, they potentiate the immune response [112]. Oncolytic viruses have been evaluated in different clinical trials, but the results have not always been promising [113]. Pexa-Vec (pexastimogene devacirepvec; JX-594) is an oncolytic virus engineered to express the transgenes human granulocyte-macrophage colony stimulating factor (GM-CSF) and beta-galactosidase [114]. The first international randomized trial on oncolytic therapy and HCC patients is the TRAVERSE study [115]. A total of 129 patients were enrolled and were randomly assigned to receive Pexa-Vec plus Best Supportive Care (BSC) or BSC alone. The results of this study demonstrated that the oncolytic virus was not able to improve the OS in HCC patients who failed sorafenib therapy. On the other hand, Pexa-Vec showed a tolerable safety profile and a satisfying degree of T-cell stimulation. It is important to highlight that this combination therapy showed a potentially synergistic mechanism of action and did not induce an increase in toxicity. Therefore, this study suggests that better results could be achieved by a combination of the oncolytic virus Pexa-Vec and another HCC immunotherapy, such as immune checkpoint inhibitors, which modulate the cancer microenvironment.

Epigenetic gene dysregulation, such as aberrant methylation or altered transcription factor binding, is strongly involved in HCC tumorigenesis [116]. Decitabine, a DNA-demethylating agent, has shown anti-cancer and immune-modulatory effects [117]. In an open-label, single-arm, phase I/II study, fifteen patients with aHCC were enrolled and treated with a lower dose of decitabine (6 mg/m^2^/day). The results showed a beneficial clinical response, prolonging the PFS and OS to 4 and 11 months, respectively; the epigenetic drug decitabine also showed a favorable adverse event profile in these patients [118].

The limitations of the DNA methyltransferase inhibitors (DNMTs) are linked to the short half-lives that reduce the in vivo efficacy and tolerability. For the purpose of overcoming the latter limitation, decitabine is used alone or in combination with other HCC therapies at a low dose.

It has been demonstrated that high histone deacetylase (HDAC) expression correlates to a higher incidence of HCC and, in the past few years, HDAC inhibitors (HDACis) have been under evaluation in clinical trials of aHCC patients [119]. In particular, the SHELTER study, an exploratory, multi-center, open-label phase I/II study, investigated the effect of the addition of Reminostat, a HDACi, to an ongoing treatment with sorafenib in patients with aHCC [120]. The results of the study showed a median PFS of 6.5 and an overall survival of 8.0 months. The treatment with Reminostat alone resulted in a median PFS of 1.8 and an OS of 4.1 months. Moreover, Reminostat alone or in combination was safe and well-tolerated at all dose levels studied. The main molecular mechanism of action of this drug is the induction of cell death in HCC patients and this effect is linked to the increased activity of caspase-3 and 9. In combination with another therapy, such as sorafenib, reminostat is able to potentiate the response to sorafenib-induced apoptosis. 

Currently, only preclinical data are available on the effect of other HDACis, such as panobinostat, vorinostat, and belinostat, on HCC cells [121,122,123].

## 8. Current Gaps and Promising Approaches

The several trials we have described testify to the great commitment of researchers to finding new therapeutic solutions for HCC. Although several antiangiogenic molecules and immune checkpoint inhibitors have been approved by the FDA for HCC treatment, there are some limitations due to the unsatisfactory efficacy of targeted therapies, the difficulty of identifying clinical biomarkers, and the difficulty of obtaining a long-lasting response to the therapy. The drugs currently available allow for an increase in survival, even though it is not many months. Furthermore, very often the quality of life of patients does not receive a clear benefit.

There are two main reasons for the lack of clear efficacy. First, the patient’s health condition is frequently suboptimal. In fact, liver cirrhosis is the background of many HCCs, whereas in the case of non-cirrhotic liver NASH–HCC the diagnosis is often late due to the lack of true screening for and the perception of low risk in NAFLD. Second, drug therapies are carried out in accordance with a protocol after the failure of other therapeutic options with advanced disease and the patient in a non-optimal state. Regarding the efficacy of drugs, it must be considered that the two main classes act as inhibitors of enzymatic cascades or receptors involved in carcinogenesis. However, these mechanisms, although important from a molecular point of view, are not the only ones responsible. This involves an incomplete action on carcinogenetic mechanisms, which have not been fully clarified. The pharmacological toxicity that is inevitably entailed in a patient who already has imperfect liver function can be given no small weight. Future therapeutic efforts will be directed to avoiding these troubles.

From this perspective, Wang et al. prepared a novel microcrystalline formulation of sorafenib (Sor-MS) and evaluated its efficacy and toxicity in mouse models [124]. The release of sorafenib into HCC tissues by injection inhibited the in vivo proliferation of HCC and the expression of epithelial–mesenchymal transition (EMT)-related factors in a long-acting manner. Moreover, compared with oral administration, it alleviated the side effects of sorafenib, avoiding damage to the capillary network of the fundus of the eye. Furthermore, a recent multi-center study showed a correlation between treatment-related adverse events and improved outcomes in patients with HCC receiving immune checkpoint inhibitors in clinical trials and in routine practice [125]. These findings could be useful to identify predictive biomarkers of toxicity and response. Another issue is the use of drug therapy as an adjuvant in surgical therapy (open and loco-regional). This modality could further enhance the beneficial effects of drugs at an early stage of disease and limit the risk of relapse. Globally, a multidisciplinary approach to HCC management, that is a combination of surgical and pharmacological interventions, will ensure the fine-tuning of a personalized therapy and an improvement in outcomes.

Concerning the HCC biomarkers, the exosomes have been confirmed to carry ncRNAs, transfer them to target cells, and bind the corresponding target molecules [126]. Furthermore, they are involved in both the proliferation and metastasis of HCC cells by promoting angiogenesis and the epithelial–mesenchymal transition (EMT) and inhibiting the function of the immune system. Moreover, the stability expressed in bodily fluids makes them the best candidates for liquid biopsy. Thus, exosomal ncRNAs have promising application prospects as biomarkers and targeted molecules for HCC therapy.

Finally, we believe that precision medicine that aims to genetically modify immune cells to destroy cancer cells may offer the best therapeutic option for the future.

## 9. Conclusions

**Figure 1:**
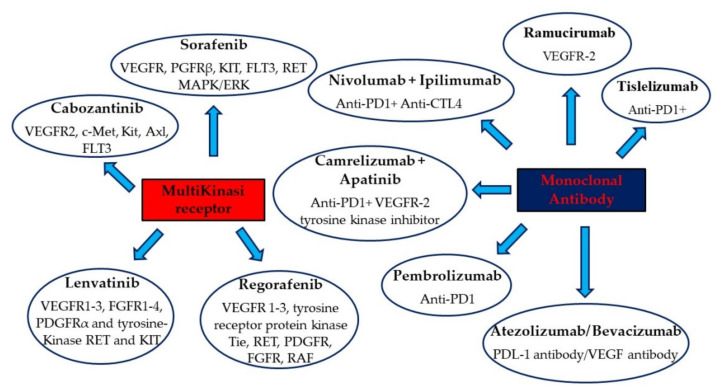
FDA-approved and main drugs from ongoing trials for HCC.

The drug therapy of HCC represents a challenge for clinicians and researchers (Figure 1). The coexistence in most cases of cirrhosis with poor health conditions does not always allow us to use all of the available therapeutic options. In cirrhotic patients, including those with zero viral load, prevention remains the most valid means of fighting HCC. Although liver transplantation and surgery can be used early on, local ablative therapy represents a valid compromise between risks and benefits in non-advanced cases.

In patients with NASH, liver function is generally better preserved, although problems related to metabolism and cardiovascular risk are often associated with this disease. In these cases, surgical therapy may represent a solution for the eradication of the disease.

For many years, pharmacological therapies have been limited to sorafenib, which has allowed for some improvement in survival but not in quality of life. Recently, lenvatinib and, in particular, the atezolizumab–bevacizumab combination have been demonstrated to increase survival in HCC patients. Other molecules under study also appear to be possible therapeutic alternatives, albeit with a considerable number of side effects. The new possible therapeutic scenarios represented by vaccines and epigenetic drugs could be the future drug therapy of HCC.

Therefore, targeted therapies for HCC are a topic of great interest and constantly being updated thanks to the numerous ongoing clinical trials.

## Figures and Tables

**Table 1:** The first and second line of therapy of HCC (Phase III trials).

Drugs	Targets	Study	Primary Endpoint
Sorafenib	VEGFR, PDGFRβ, FLT3, RET	SHARP [45]	OS
Lenvatinib	VEGFR1-3, FGFR1-4, PDGFRα, RET, KIT	REFLECT [56]	OS
Atezolizumab/ Bevacizumab	PDL-1 antibody/VEGF antibody	IMbrave150 [61]	OS
Regorafenib	VEGFR 1–3, tyrosine receptor protein kinase Tie, RET, PDGFR, FGFR, RAF	RESORCE [70]	OS
Cabozantinib	VEGFR2, c-Met, Kit, Axl, FLT3	CELESTIAL [74]	OS
Ramucirumab	VEGFR-2	REACH [76]	OS

VEGFR, vascular endothelial growth factor receptor; PDGFRβ, platelet-derived growth factor receptor beta; FLT3, fms-like tyrosine kinase-3; FGR, basic fibroblast growth factor receptor; OS, overall survival.

**Table 2:** FDA-approved drugs and ongoing trials for HCC.

Drugs	Targets	Study	Primary Endpoint
Nivolumab + Ipilimumab	Anti-PD1 + Anti-CTL4	CHECKMATE 040 (ongoing multi-center, multiple parallel cohort, open-label clinical trial) [71]	ORR
Camrelizumab + Apatinib	Anti-PD1 + VEGFR-2 tyrosine kinase inhibitor	RESCUE (nonrandomized, open-label, Phase II trial) [88]	ORR
Pembrolizumab	Anti-PD1	KEYNOTE 240 (randomized, double-blind, Phase III trial) [87]	OS and PFS
Tislelizumab vs. Sorafenib	Anti-PD1 + Multi-kinase inhibitor	RATIONALE 301 (global, Phase III, randomized, open-label, multi-center study) [89]	OS between two treatment groups

VEGFR, vascular endothelial growth factor receptor; ORR, overall response rate; OS, overall survival; PFS, progression-free survival; +, in combination with; vs, compared with.

## Data Availability

Not applicable.
